# Risk Prediction Models for Ischemic Cardiovascular Outcomes in Patients with Acute Coronary Syndrome

**DOI:** 10.31083/j.rcm2404106

**Published:** 2023-04-13

**Authors:** Qi Zhang, Jie Gao, Xiaoying Yin, Song Zhang, Yifan Wang, Hongmei Ji, Xiao Zhang, Dongli Song, Jiali Wang, Yuguo Chen

**Affiliations:** ^1^Department of Emergency Medicine, Qilu Hospital of Shandong University, 250012 Jinan, Shandong, China; ^2^Shandong Provincial Clinical Research Center for Emergency and Critical Care Medicine, Institute of Emergency and Critical Care Medicine of Shandong University, Chest Pain Center, Qilu Hospital of Shandong University, 250012 Jinan, Shandong, China; ^3^Key Laboratory of Emergency and Critical Care Medicine of Shandong Province, Key Laboratory of Cardiopulmonary-Cerebral Resuscitation Research of Shandong Province, Shandong Provincial Engineering Laboratory for Emergency and Critical Care Medicine, Qilu Hospital of Shandong University, 250012 Jinan, Shandong, China; ^4^The Key Laboratory of Cardiovascular Remodeling and Function Research, Chinese Ministry of Education, Chinese Ministry of Health and Chinese Academy of Medical Sciences, The State and Shandong Province Joint Key Laboratory of Translational Cardiovascular Medicine, Qilu Hospital of Shandong University, 250012 Jinan, Shandong, China

**Keywords:** acute coronary syndrome, ischemic events, risk score, prognosis, mortality

## Abstract

Acute coronary syndrome (ACS) has a high incidence of adverse cardiovascular events, even after early invasive treatment. Patients may still have a poor prognosis after discharge. The keys to the long-term survival of patients with ACS include effective treatment in a timely manner and identification of those patients who are at higher risk for long-term adverse events. Therefore, several nations have now devised a range of risk assessment models to provide data for accurately formulating treatment plans for patients with various risk levels following an ACS to prevent short and long-term cardiovascular events. The purpose of this article is to review the risk scores associated with mortality and ischemic events in patients with ACS. By using the clinical risk prediction score, we can accurately and effectively judge the prognosis of patients, so as to take a more reasonable treatment.

## 1. Introduction

The global burden of cardiovascular disease (CVD) remains high. Although the age-related standardized mortality rate of CVDs has decreased in the past decade, the absolute number of deaths caused by CVDs has increased by 12.5%. Compared with other types of cardiovascular diseases, the manifestations and adverse outcomes of acute coronary syndrome (ACS) are generally recognized. More than 2 million people die of ACS each year in the United States, while the annual death toll in Europe and North Asia is much higher [1]. Due to the increased use of invasive strategies, the development of antiplatelet and anticoagulant medications, the optimization of secondary prevention strategies such as statins, and especially the improvement of risk stratification, the mortality of patients has decreased following ACS. However, there is still a substantial risk of recurrent adverse cardiovascular events [2, 3, 4]. Since the risk of new or recurrent ischemic cardiovascular events and death is heterogeneous in these patient populations, it is important to assess the risk and weigh the potential benefits of currently available therapies, regardless of long or short-term follow-up [5, 6, 7, 8].

Therefore, it important to develop risk models to help stratify the early risk of ACS and to select appropriate treatment strategies to prevent new or recurrent adverse events. The occurrence and mortality of adverse cardiovascular events in patients with ACS is influenced by a variety of variables. Several risk assessment models for patients with ACS based on different risk factors have now been developed to help clinicians better risk stratify these patients [9, 10, 11, 12]. These predictive models have now been included in the clinical guidelines for the treatment management of patients with ACS [13, 14]. The prognosis of individuals with ACS is reviewed in this article along with the most recent risk prediction algorithms.

## 2. Risk Scores of Patients with ACS and New Progress after Combination with Biomarkers

### 2.1 The Global Registry of Acute Coronary Events (GRACE) Risk Score

The GRACE risk score accurately predicts the likelihood of in-hospital death and 6-month all-cause death and nonfatal myocardial infarction composite endpoint events in patients with ACS. The model has good predictive ability, with a C statistic of 0.85. It also performs well in two external validation sets. With the increase of risk score, the in-hospital mortality increased [11, 15]. This scoring system is derived from the largest ACS registration study in the world [16]. However, it contains many variables which may not be readily available at the time of admission [17]. In the GRACE risk score (2.0) proposed in 2014, the admission Killip classification and serum creatinine were modified to include the use of diuretics, making the initial data easier to obtain. The C index of death, which can predict both short and long-term mortality, exceeds 0.82 in the overall population at 1 and 3 years when using the French registry of Acute ST-elevation and non-ST-elevation Myocardial Infarction (FAST-MI) 2005 cohort [18]. Another study cohort also confirmed the good predictive ability of this scoring system [19]. Because the GRACE risk model was derived nearly 20 years ago, fewer Asians were initially included in the early database. However, in subsequent studies, the GRACE scores conducted in Asian populations also showed good predictive power, which further confirmed that despite the advancements in modern therapy and management, GRACE scores continue to accurately classify patients with ACS [20, 21, 22].

Although biomarkers have now been included in risk scores, several studies have not confirmed that high sensitive cardiac troponin (hs-cTn) and B-type natriuretic peptide (BNP) can improve the GRACE score [17]. However, a recent study showed that the area under the curve (AUC) of the GRACE risk estimate after growth differentiation factor 15 (GDF-15) adjustment increased from 0.79 to 0.85 (*p *< 0.001) in the validation cohort using biomarkers. The GRACE score was also enhanced once the N-terminal fragment brain natriuretic peptides (NT-proBNP) were included [23]. Investigating the value of biomarkers in clinical risk scores needs to be further explored.

### 2.2 Thrombolysis in the Myocardial Infarction (TIMI) Risk Score

The TIMI risk score was first proposed in 2000. It performs well in terms of risk stratification for the prediction of 30-day mortality in ST segment elevation myocardial infarction (STEMI) patients after admission. In addition, the TIMI score in patients with unstable angina/non-ST segment elevation myocardial infarction (UA/NSTEMI) also performs well in patients seen in the emergency room with chest pain. The variables included in these two scoring systems are mainly derived from the data and electrocardiogram (ECG) that are easily available in the emergency room. These indicators are good at predicting short-term prognosis and long-term major adverse cardiovascular events (MACE) [24, 25, 26, 27]. It has been suggested to use a modified TIMI score (mTIMI, range 0–10) which gives some variables more weight. The goal was to determine whether improving the risk stratification of the TIMI risk score by giving more weight to ischemic ECG abnormalities and troponin elevations could help to more safely discharge patients following 12-hour troponin testing. While the mTIMI risk score outperformed the original score (AUC 0.87 versus 0.77, *p *< 0.001), neither score by itself is sensitive enough at scores >0 to permit early and safe discharge without follow-up care. Therefore, in patients with normal ECG and negative troponins, the utility of TIMI and mTIMI scores for risk classification is limited [28, 29, 30].

A study proposed a prediction model for the prognosis evaluation of a new antiplatelet drug based on the TIMI risk score. Vorapaxar, a new antiplatelet drug, can be effectively used for secondary prevention of stable patients with identified atherothrombosis [31, 32, 33]. To evaluate the safety and effectiveness of vorapaxar in secondary prevention of patients with an acute myocardial infarction (AMI) using the results of the thrombin receptor antagonists in the secondary prevention of atherosclerotic thromboischemic events and myocardial infarction hemolysis (TIMI50) test, a TIMI risk score for secondary prevention (TRS2°P) score was developed to predict long-term recurrent cardiovascular (CV) events [34, 35]. This score is effective in determining the likelihood of repeat MACE in various risk categories, and shows a high predictive ability in external validation. After patients are stratified according to the TRS2°P, high risk groups such as elderly patients with myocardial infarction and patients with complications, can also benefit from the treatments recommended by guidelines such as antiplatelet medication [36, 37]. Patients with a high risk of recurrent CV events often have complications following anti-platelet medication and invasive therapy. Careful follow-up is required in these patients to minimize future MACE and will be the subject of future studies in these high-risk ACS patients.

Although the TIMI risk score is a powerful tool which can be used for risk stratification of ACS patients in the emergency room, it should not be used as the only means to determine the disposition of patients. Studies have found that NT-proBNP outperforms the TIMI score in predicting death following an AMI [9, 38]. In addition, the combination of baseline NT-proBNP, C-reactive protein, creatinine level, and inflammatory markers in the TIMI risk score provides more data regarding risk stratification and prognosis following an ACS [39, 40, 41].

Recently, a biomarker-based risk model for MACE within one year after admission for Chinese patients with ACS was proposed, which highlights the significant role of NT-proBNP in predicting MACE in ACS patients. The final model combines NT-proBNP with six other clinical variables, and showed better discrimination in the validation cohort (C statistic 0.79, 95% CI 0.73–0.85), and was superior to the GRACE and TIMI risk scores [42]. The European Society of Cardiology (ESC) now recommends that NT-proBNP be used as a prognostic factor for patients with coronary artery disease (Class IIa, level B) [43]. NT-proBNP is a neurohormone peptide secreted by ventricular cells and is closely related to ventricular dysfunction. Future studies will continue to define the role of NT-proBNP in risk stratification of ACS patients in clinical practice.

### 2.3 Age, Creatinine and Ejection Fraction (ACEF) Score

The ACEF score is a straightforward and practical risk prediction technique since it only includes three independent criteria. It was initially developed for patients undergoing elective heart surgery to assess perioperative mortality. In order to compute the ACEF score, the following formula was used: age (years) / ejection fraction (%) + 1 (in case serum creatinine values were >2 mg/dL). Studies have confirmed its accuracy compared to more complicated risk scores [44]. Subsequent studies showed that the ACEF score also had important prognostic value for patients with ACS. A cohort study validated the ACEF score, and demonstrated that these three factors could independently predict the outcome of patients with ACS following coronary revascularization and produce a predictive value comparable to the GRACE score [10]. The ACEF score has an independent predictive effect on 1-year mortality with a strong AUC of 0.79, according to data from the Korean Acute Myocardial Infarction Registry, which included data from an ACS cohort undergoing percutaneous coronary intervention (PCI) [45]. When applied to patients with a non-ST-elevation ACS (NSTE-ACS), it demonstrated superior discrimination compared to other complex risk stratification models [46].

In addition, other risk factors have been added to the ACEF score and it has been combined with other scores to further enhance its performance. The carotid plaque score (cPS) was assessed with data from carotid ultrasonography. By combining cPS with the modified ACEF score, the degree of freedom of MACE was 71% and 31% (*p *< 0.001) for lower and higher scores at 5 years. When combined with ordinary ACEF scores in ACS, cPS enhances predictive values [47]. When diabetes, a common risk factor for patients with coronary heart disease, is included in the new ACEF diabetes comprehensive score, better accuracy and calibration factors are achieved [48].

Many complications other than cardiovascular adverse events can also be predicted by the ACEF risk score. In a study, the ACEF score accurately predicted additional clinical outcomes, such as bleeding, in addition to in-hospital mortality [49]. In patients with myocardial infarction who have ST segment elevation after a coronary intervention, a high ACEF score predicted the occurrence of contrast-induced acute kidney damage (CI-AKI) [50]. It was also reported that the ACEF score achieves good performance in identifying the adverse prognosis of high-risk patients with complex coronary lesions after PCI, including bifurcation lesions and chronic total occlusions [51, 52].

One of the variables included in the ACEF score, the ejection fraction, will change with the degree of myocardial ischemia, so that the timing of evaluating the ACEF score is particularly important. These studies have demonstrated that the ACEF score can provide a new and simple tool for daily clinical practice to stratify the risk of patients with ACS. Despite the fact that the ACEF score is simple to use and has performed on par with more complex models, long-term validation studies in various populations, hospitals, and nations are still required to assess its role in ACS patients.

## 3. Simple Risk Scores for Short-Term Prognosis of Patients with ACS

### 3.1 The Canada Acute Coronary Syndrome (C-ACS) Risk Score 

Although many studies have proposed a variety of prognostic risk scores for ACS, an appropriate score for patients admitted for the first time with ACS still needs to be developed. Based on the data from patients with AMI from ACS-1 registries in Quebec and Canada, a C-ACS risk score has been developed, and verified in patients with ACS in four large data sets. The C-ACS score, which varied from 0 to 4, was generated using logistic regression modeling. One point was given for each of the following variables: age 75 years, Killip >1, systolic blood pressure 100 mmHg, and heart rate >100 beats per minute. This score has a C statistical value of 0.79. Notably, when the C-ACS score is 0, there is a potential to accurately identify 97% of short-term survivors, and to evaluate the possibility of in-hospital death in ACS patients [53]. In one study, the C-ACS score outperformed age in predicting in-hospital mortality among patients with AMI [54]. Some studies have shown that not only does the C-ACS risk score perform well in predicting infections that may occur following PCI in patients with AMI, the data suggests that these patients are more prone to develop contrast-induced nephropathy after PCI when the C-ACS risk score increases [55, 56]. This score can be obtained by calculating a number of variables that are not based on blood tests and ECG interpretations. It performs well for predicting both hospital and long-term death, and can be easily calculated, making it better suitable for diagnosing and treating ACS in the emergency department as well as early risk stratification following admission.

### 3.2 Portuguese Registry of Acute Coronary Syndromes (ProACS) Risk Score

Over 45,000 patients from the Portuguese ACS Registry were included in the study of the ProACS risk score [57]. In all the research cohorts as well as independent external validation cohorts, the score has satisfactory discrimination ability [58, 59]. The ProACS risk score does not perform well in predicting long-term prognosis compared to the GRACE risk score [11]. Although in this study, the ProACS had a high recognition rate and a similar C-statistic compared with the most effective risk stratification score, it was under calibrated in the NSTE-ACS cohort. The ProACS risk score was derived to facilitate the use of instant information to help determine early risk stratification after admission, to assist in making timely and effective decisions. Therefore, it mainly focuses on short-term results, while the results of long-term follow-up are insufficient, and the included variables are limited.

### 3.3 Cardiovascular Disease in the China-Acute Coronary Syndrome (CCC-ACS) Risk Score

According to the baseline data of 62,546 unselected patients with ACS from multiple hospitals in China, the CCC-ACS score was recently developed to predict in-hospital mortality in these ACS patients. This score differs from the China acute myocardial infarction (CAMI) risk score proposed in 2018, which has the same forecasting ability as the GRACE score, but it contains up to 16 variables, and only the computational complexity reduces its applicability [60]. The CCC-ACS score includes seven variables, which are different from other risk scores. The variables included in the CCC-ACS score take into account the evaluation of patients before blood testing. Except for ST segment changes on the ECG, the other variables mainly focus on the patient’s vital signs and medical history. The AUC of this new risk score in the training dataset was 0.84 (Hosmer-Lemeshow goodness-of-fit test *p* = 0.1), and it also performed well in the validation dataset [61]. These two scoring models have the drawback that the research population is close to 100% non-white and exclusively Asian, which prevents them from being really generalized until they are further validated in diverse populations and nations.

## 4. Risk Scores for Specific Population or including Special Examination

### 4.1 The Cardiovascular Magnetic Resonance (CMR) Risk Score

CMR is a valuable tool for determining the risk of heart failure. It characterizes myocardium by using a variety of different imaging parameters, which has been widely accepted as a reference standard for quantifying chamber size and ejection fraction [62]. The predictive ability of CMR in myocardial infarction (MI) is constantly being explored. At present, some studies have confirmed its role in AMI and other special types of myocardial infarction, such as unrecognized myocardial infarction (UMI) and myocardial infarction with nonobstructed coronaries (MINOCA) [63, 64, 65, 66]. Recently, the CMR risk score was proposed for risk categorization in STEMI patients. It includes left ventricular ejection fraction (LVEF), microvascular occlusion (MVO) and myocardial infarction (MI) size. In the derivation cohort, the score performed well in predicting the 1-year composite endpoint, and even exceeded the GRACE score [67]. Compared to GRACE and transthoracic echocardiography-LVEF, the CMR score offers additional prognostic classification and may have an impact on how patients with STEMI are managed [68]. For patients with an STEMI with an LVEF <50% by echocardiography, selective use of CMR can significantly improve the prediction of MACE [66]. In addition, a study has shown that in all time periods, CMR had a similar predictive value for the main endpoint [69]. The extrapolation of this risk scoring model still needs to be verified in larger multicenter research cohorts.

### 4.2 The SILVER-AMI (Comprehensive Evaluation of Risk in Older Adults with AMI) Mortality Risk Score

Compared to young patients, elderly patients with AMI have more comorbidities, and an increased risk of death following an AMI may result from poorer physiological reserves and more dysfunction (including physical ability and cognition). Functional decline has been linked to limitation in mobility as a potential mechanism [70, 71]. In a study of 3006 patients ≥75 years old who survived an AMI after discharge, Dodson and colleagues proposed a model to predict 6-month mortality risk in this population, the SILVER-AMI mortality risk model, which included around 9.5% of the patients who were non-white. In addition to the more common clinical features, the final risk model also includes four features specifically designed for the elderly: hearing loss, poor mobility, weight loss and poor health as reported by patients. The model has a good capacity for discrimination (AUC of the validation queue = 0.84) and is properly calibrated (Hosmer-Lemeshow *p *> 0.05) [72, 73, 74]. Using this scoring model, a 180-day readmission risk model for elderly patients with AMI was established. The functional mobility of patients is also emphasized in the variables included in this risk model. Similar information is obtained in the verification queue, where the model’s differentiation ability is 0.68. In addition, over 40% of participants were hospitalized after 180 days following an AMI [75]. This work was the first to provide a mortality risk model for senior patients following discharge from the hospital with an AMI. However, the study still has some limitations. First, although the study was fully validated internally, the performance of this prediction model was not centrally evaluated in the external database. In addition, the access to information about related dysfunction needed to be evaluated by this score, is relatively limited. It is worth noting that the elderly often have multi organ and multi system chronic diseases, which may lead to inaccurate reporting of the cause of death. A summary of main risk models for determining the prognosis of ACS are provided in Table 1 (Ref. [11, 15, 18, 24, 42, 53, 58, 60, 61]).

**Table 1.** **Summary of main risk models for the prognosis of acute coronary syndrome**.

Items	Published year	Originated study	Population	Primary outcome	Predictor variables	Modeling method/C-index
TIMI risk score [24]	2000	Derivation cohort: unfractionated heparin group in TIMI 11B trial (n = 1957)	NSTE-ACS	The composite of all-cause death, new or recurrent MI, severe recurrent ischemia requiring urgent revascularization within 14 days	Age 65 years or older, at least 3 risk factors for CAD, significant coronary stenosis, ST-segment deviation, severe angina symptoms, use of aspirin at least of 7 days, initial cardiac enzyme elevation	Logistic regression/Derivation cohort: 0.65
		Validation cohort: enoxaparin group in TIMI 11B trial (n = 1953), unfractionated heparin group in ESSENCE trial (n = 1564) and enoxaparin group in ESSENCE trial (n = 1607)				Validation cohort: 0.63
GRACE risk score [11]	2003	Derivation cohort: GRACE registry (n = 11,389)	ACS	In-hospital all-cause death	Age, cardiac arrest at hospital arrival, Killip class, heart rate, systolic blood pressure, ST-segment deviation, initial circulating creatinine, initial cardiac enzyme elevation	Logistic regression/Derivation cohort: 0.83
		Validation cohort: subsequent cohort of GRACE registry (n = 3972), GUSTO-IIb trail (n = 12,142) *				Validation cohort: 0.84 and 0.79
GRACE risk score [15]	2006	Derivation cohort: GRACE registry (n = 21,688)	ACS	All-cause death or the composite of all-cause death and MI over 6 months	Age, cardiac arrest at hospital arrival, Killip class, heart rate, systolic blood pressure, ST-segment deviation, initial circulating creatinine, initial cardiac enzyme elevation	Cox regression/Derivation cohort: 0.82 for death, 0.70 for death/MI
		Validation cohort: Subsequent cohort of GRACE registry (n = 22,122), GUSTO-IIb trail (n =12,142)				Validation cohort: 0.82 for death, 0.73 for death/MI
C-ACS risk score [53]	2013	Derivation Cohort: n = 6182	STEMI	In-hospital or 30-day and 1- or 5-year all-cause mortality	Age, initial systolic blood pressure (SBP), and initial heart rate (HR), Killip class	Logistic regression/Validation Cohort: 0.79 for in-hospital death
		AMI-QUEBEC (n = 1555) *	NSTE-ACS			
		Canada ACS-1 registry (n = 4627)				
		Validation Cohort: n = 23,310				
		Canada ACS-2 registry (n = 1956)				
		Canada-GRACE (n = 10,195) *				
		EFFECT-1 (n = 11,159) *				
GRACE 2.0 risk score [18]	2014	Derivation Cohort: GRACE registry (n = 32,037)	ACS	1-year and 3-year mortality, and death/MI, overall and in hospital survivors	Age, systolic blood pressure, pulse, creatinine, Killip class	Cox regression/Validation cohort: 0.82 for death, 0.78 for death/MI
		Validation Cohort: FAST-MI 2005 (n = 3059)				
ProACS risk score [58]	2017	Derivation Cohort: randomly separated 60% of the first 31,829 patients (n = 17,380)	ACS	All-cause mortality during the index hospitalization	Systolic blood pressure, Killip class, ST-segment elevation, age	Logistic regression/Derivation cohort: 0.80
		Validation Cohort: Internal validation cohort: the remaining 40% patients (n = 11,548)				Internal validation cohort: 0.79
		External validation cohort: the last 8586 patients included in the registry (n = 8532)				External validation cohort: 0.82
The CAMI score [60]	2018	Derivation Cohort: CAMI registry (n = 17,563)	ACS	All-cause in-hospital death	Age, gender, body mass index, systolic blood pressure, heart rate, creatinine level, white blood cell count, serum potassium, serum sodium, ST-segment elevation on ECG, anterior wall involvement, cardiac arrest, Killip classification, medical history of hypertension, medical history of hyperlipidemia and smoking status	Logistic regression/Derivation cohort: 0.83
		Validation Cohort: CAMI registry (n = 5854)				Validation cohort: 0.84
CCC-ACS risk score [61]	2021	Derivation Cohort: A training dataset (n = 43,774)	ACS	In-hospital death	Age, systolic blood pressure, cardiac arrest, insulin-treated diabetes mellitus, history of heart failure, severe clinical conditions (acute heart failure or cardiogenic shock), and electrocardiographic ST-segment deviation	Logistic regression/Derivation cohort: 0.84
		Validation Cohort: A validation dataset (n = 18,772)				Validation cohort: 0.85
BIPass risk model [42]	2022	Derivation Cohort: BIPass registry (n = 4407)	ACS	MACE which was defined as the composite of cardiac death, new or recurrent MI, and ischemic stroke after enrollment through 12 months	Age, hypertension, previous myocardial infarction, stroke, Killip class, heart rate, and NT-proBNP	Cox regression/Derivation cohort: 0.81
		Validation Cohort: BIPass registry (n = 1409)				Validation cohort: 0.79

* GUSTO-IIb trial cohort was used for externally validation for GRACE risk score predicting 6-month all-cause death outcome. ACS, acute coronary syndrome; NSTE-ACS, non-ST-elevation ACS; STEMI, ST segment elevation myocardial infarction; ECG, electrocardiogram; MACE, major adverse cardiovascular events; AMI-QUEBEC, Acute Myocardial Infarction in Quebec; Canada-GRACE, Global Registry of Acute Coronary Events; EFFECT-1, Enhanced Feedback for Effective Cardiac Treatment; FAST-MI, the French registry of Acute ST-elevation and non-ST-elevation Myocardial Infarction.

## 5. Conclusions

Although both the GRACE and TIMI scores still need to be improved, they are widely used clinically and have strong data support. Many new risk scores are compared to the GRACE score for a very long time, which partly reflects its continuous popularity. In addition to being derived from the biggest ACS registry in the world and applying to a wide spectrum of patients, GRACE score data also has a generally flawless review system. Because of these qualities, it is better suited for assessing the long-term prognosis of ACS patients. At the same time, it also incorporates a large number of prognostic factors that are excluded from many other risk scores. However, its complexity as a in-hospital risk score occasionally restricts the use scenarios and time. Therefore, it is important to pick a risk score model with straightforward variables and strong evaluation capabilities. Here, we suggest using the ProACS risk score to forecast in-hospital mortality. Its initial positioning at the time of establishment, in addition to the qualities listed above, is to forecast in-hospital mortality. A significant issue is how to accomplish more accurate risk classification at the first time after admission in many situations due to the quick and severe condition of ACS patients. Although the score still needs to be validated in more populations and countries, its efficacy as a risk score for predicting in-hospital mortality has been confirmed. By using risk stratification, doctors can identify high-risk patients at discharge, institute invasive procedures as early as possible, and shorten the time of hospitalization in patients with low risk, reducing medical costs, and benefit patients’ physically and mentally. A good risk score should not only perform well in both simultaneous assessments of the short and long-term prognosis for ACS patients, but also be simple to use. A growing number of biomarkers have been identified that affect prognoses in ACS patients. Can we explore a simpler and more applicable score from this perspective? Of course, it is not in accordance with medical laws to promote a unified score applicable to all ACS patients, the huge differences of diseases in different regions and races cannot be ignored. Although the treatment and out of hospital secondary prevention management strategies of contemporary ACS patients are constantly improving, the existing risk models are still exploring new possibilities to play a greater role in evaluating prognosis and instituting the most effective treatment strategies to reduce both in-hospital and long-term MACE.
